# Supplementary figures and images for: Diversity and prevalence of zoonotic infections at the animal-human interface of primate trafficking in Peru
Source: PLoS One. 2024 Feb 7;19(2):e0287893. doi: 10.1371/journal.pone.0287893 (PMC10849265; doi:10.1371/journal.pone.0287893)

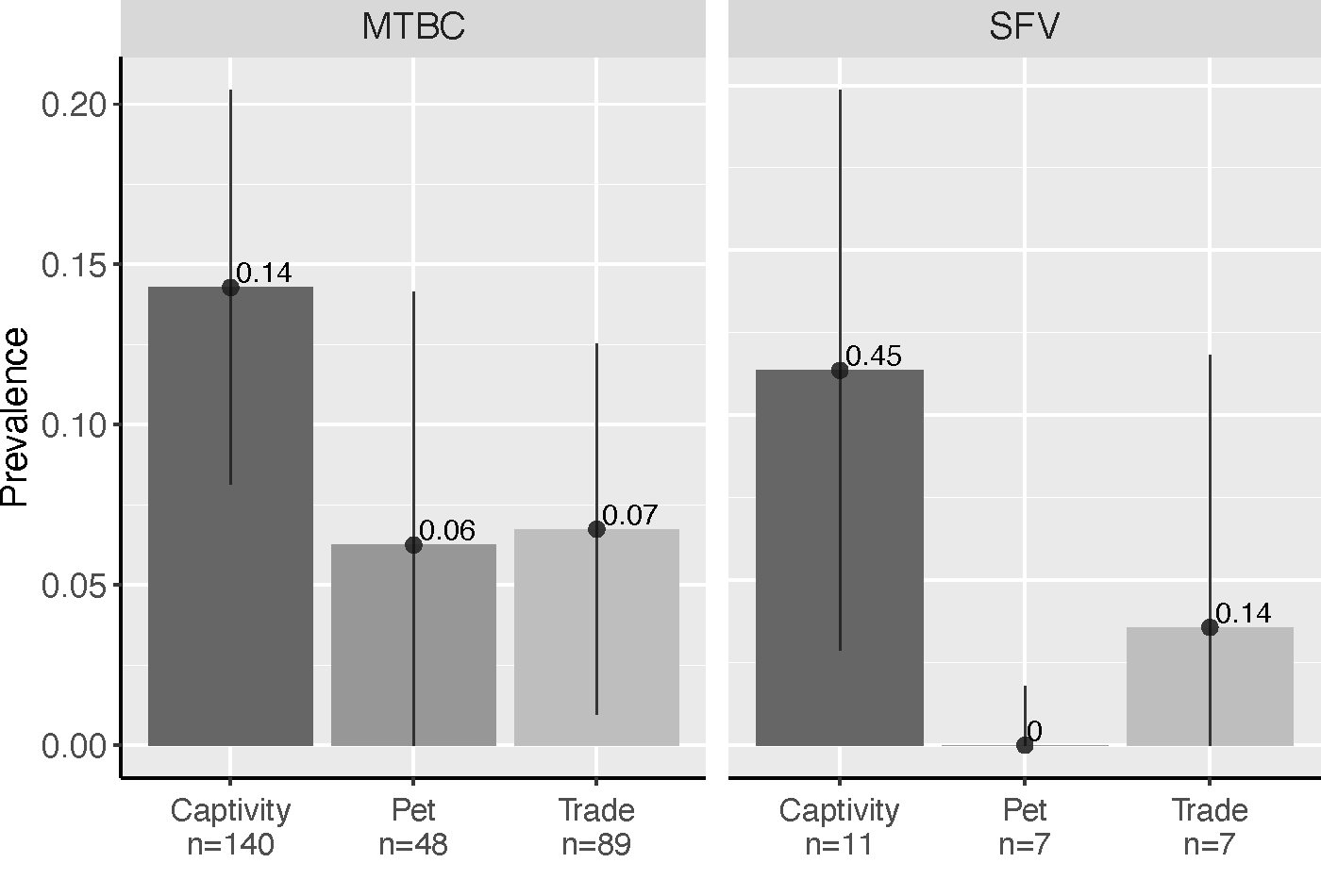

Supplement: S1 Fig — Bar plot showing the proportion of monkeys with positive status for MTBC and SFV at each context. (TIF) [file pone.0287893.s001.tif]

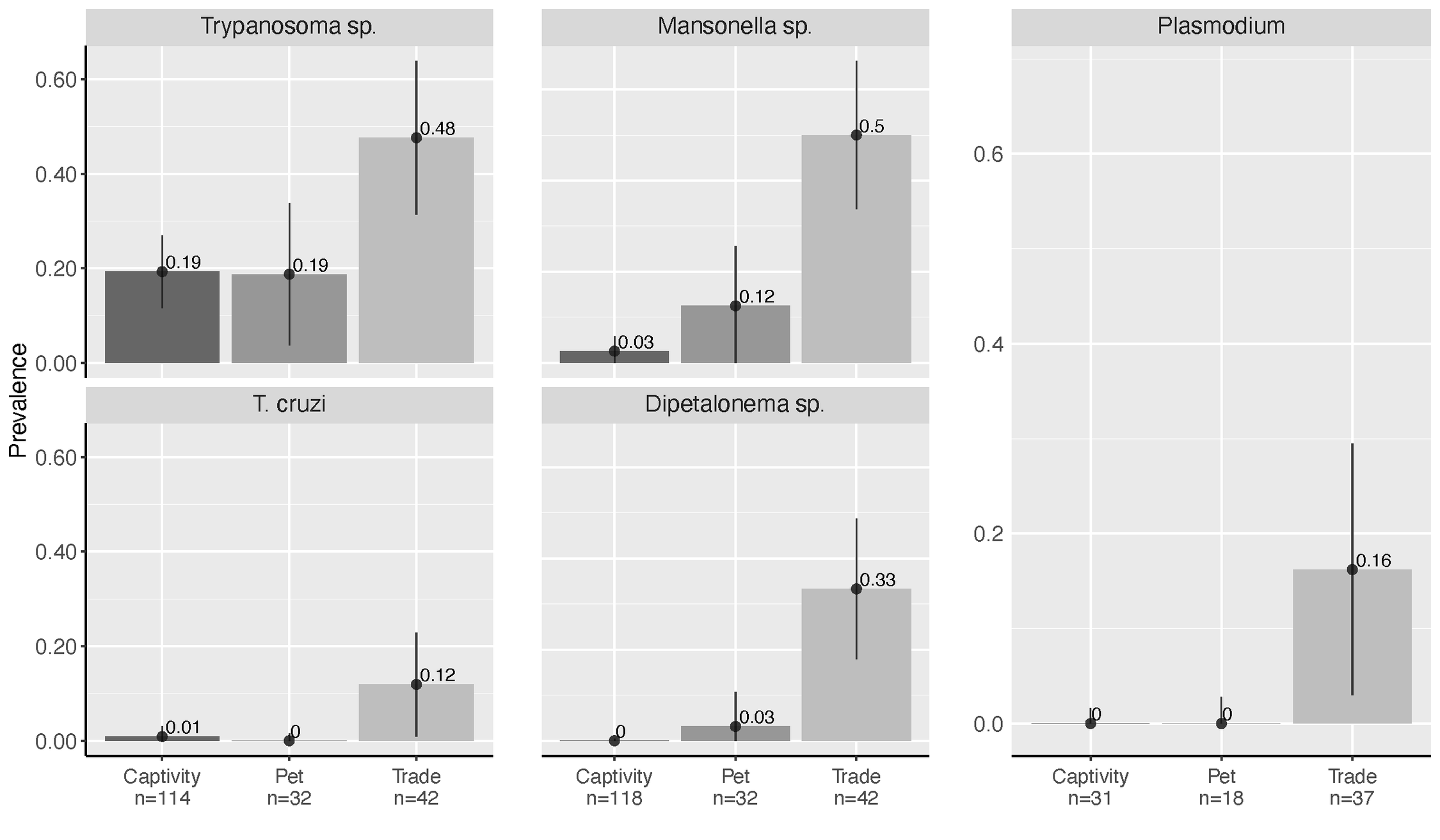

Supplement: S2 Fig — Bar plot showing the proportion of monkeys with positive status for Trypanosoma sp., Mansonella sp., tryopanosoma cruzi, Dipetalonema sp., and Plasmodium malaria/brasilianum and SFV across contexts. (TIF) [file pone.0287893.s002.tif]

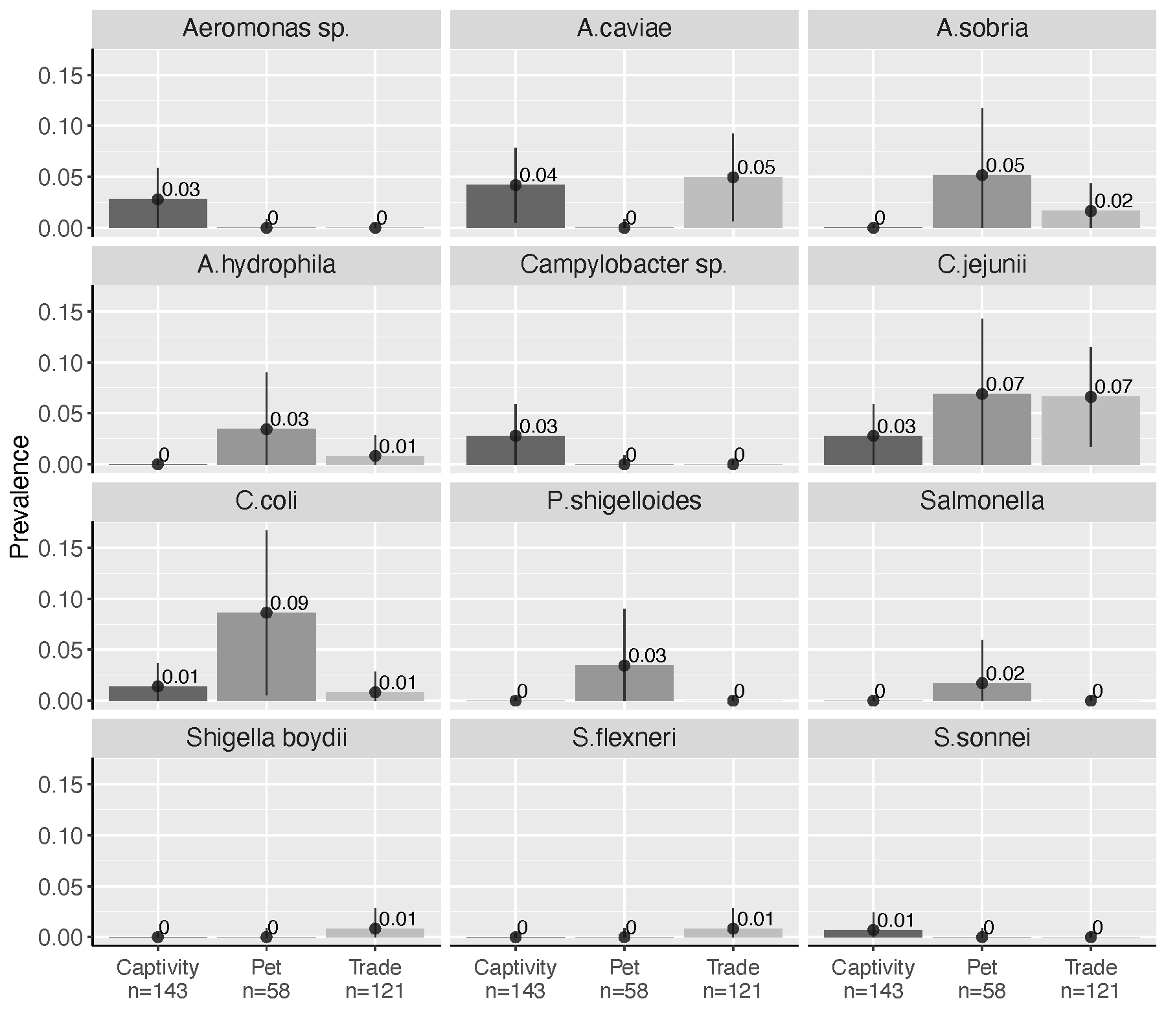

Supplement: S3 Fig — Bar plot showing the proportion of monkeys with positive status for Aeromonas sp., Aeromonas caviae, Aeromonas sobria, Aeromonas hydrophila, Campylobacter sp., Campylobacter jejunii, Campylobacter coli, Plesiomonas shigelloides., Salmonella sp., Shigella boydii, Shigella flexneri, and Shigella sonnei across contexts. (TIF) [file pone.0287893.s003.tif]

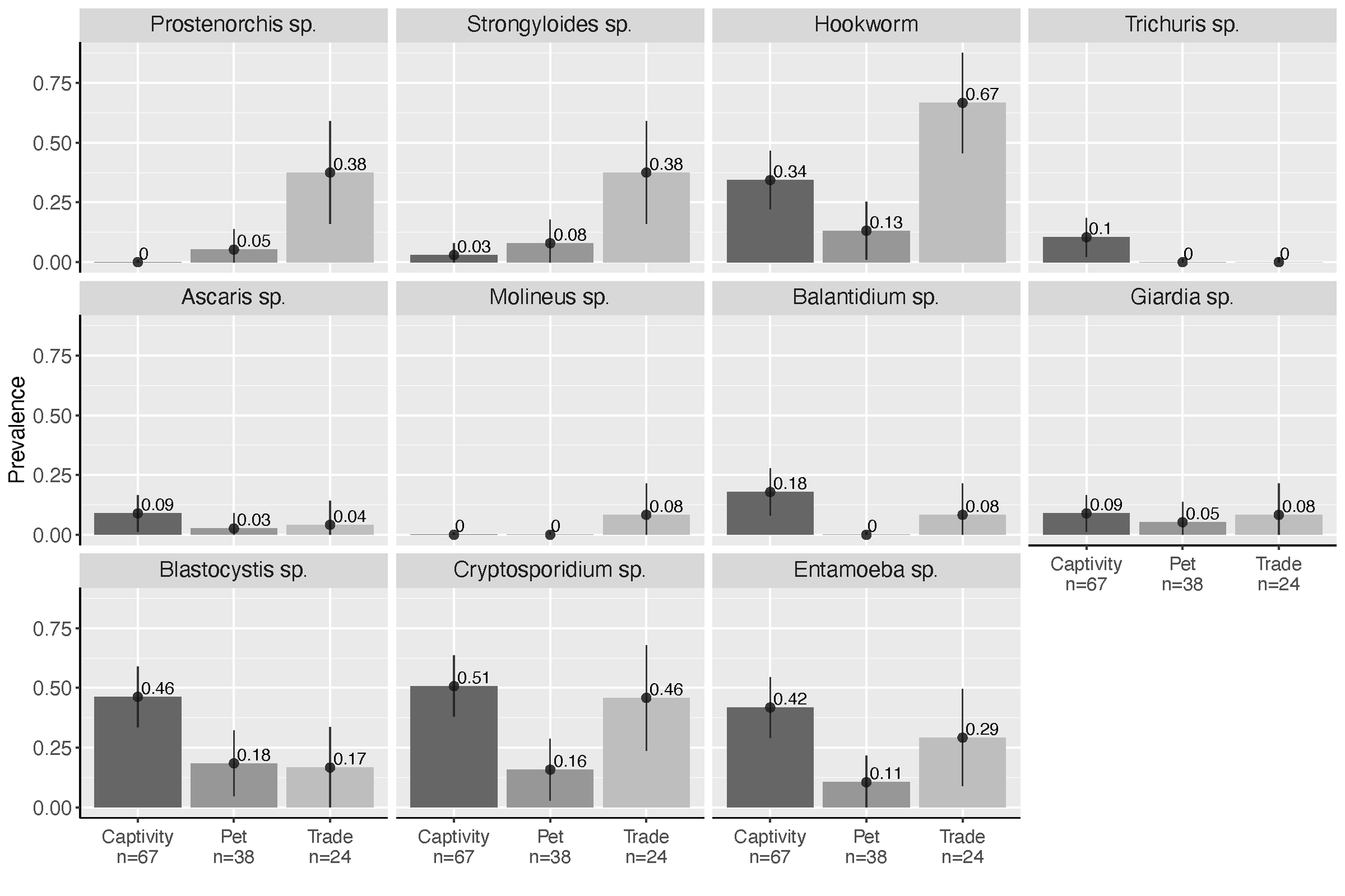

Supplement: S4 Fig — Bar plot showing the proportion of monkeys with positive status for Prostenorchis sp., Strongyloides sp., hookworms, Trichuris sp., Ascaris sp., Molineus sp., Balantidium sp., Giardia sp., Blastocystis sp., Cryptosporidium sp., and Entamoeba sp. across contexts. (TIF) [file pone.0287893.s004.tif]

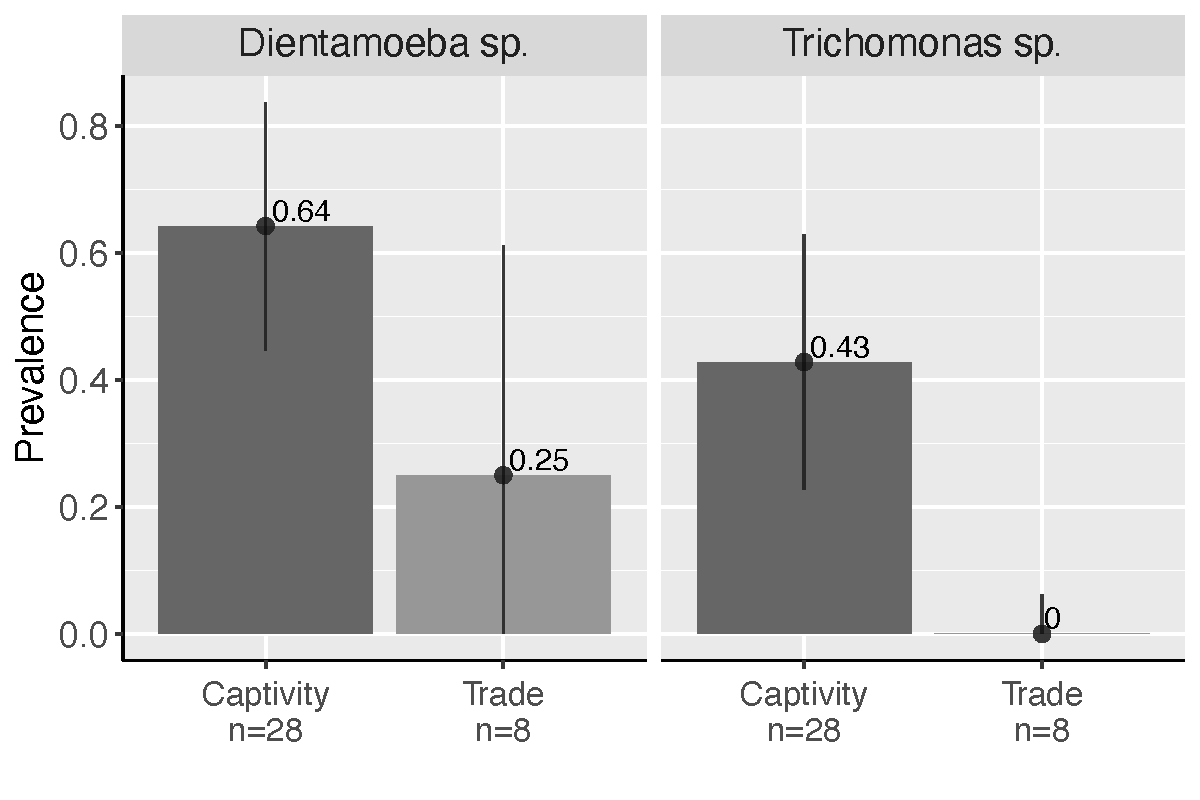

Supplement: S5 Fig — Bar plot showing the proportion of monkeys with positive status for Dientamoeba sp. and Trichomonas sp. in the trade and at captivity contexts. (TIF) [file pone.0287893.s005.tif]
