# Supplementary material for: Diversity and prevalence of zoonotic infections at the animal-human interface of primate trafficking in Peru
Source: PLoS One. 2024 Feb 7;19(2):e0287893. doi: 10.1371/journal.pone.0287893 (PMC10849265; doi:10.1371/journal.pone.0287893)
Supplement: S1 Table — This table shows the frequency of detection and prevalence of each parasite type (MTBC, SFV, hemoparasites, enteric bacteria, enteric helminths, enteric protozoa, and trichomonads) among the contexts for animal-human interaction (captivity, pet, and trade) in which trafficked monkeys are found in Peru and the results of the chi-squared test comparing the homogeneity of proportions between contexts. (DOCX) [file pone.0287893.s006.docx]

**Table S1. Frequency (Freq.) and prevalence (Prev.) of zoonotic parasites in captive monkeys found at different contexts for animal-human interaction in Peru.**

| **Parasite type** | **Captivity** | |  | **Pet** | |  | **Trade** | |  | **p-value**^**^ |
| --- | --- | --- | --- | --- | --- | --- | --- | --- | --- | --- |
|  | **Freq.** | **Prev.**  **(95% C.I)^*^** |  | **Freq.** | **Prev.**  **(95% C.I)** |  | **Freq.** | **Prev.**  **(95% C.I)** |  |  |
| MTBC | 20/140 | 14.3  (8.1-20.4) |  | 3/48 | 6.3  (0.0-14.1) |  | 6/89 | 6.7  (1.0-12.5) |  | 0.116 |
| SFV | 5/11 | 45.5  (11.5-79.4) |  | 0/7 | 0.0  (0.0-7.1) |  | 1/7 | 14.3  (0.0-47.4) |  | 0.111 |
| Hemoparasites | 25/119 | 21.0  (13.3-28.7) |  | 9/32 | 28.1  (11.0-45.3) |  | 39/42 | 92.9  (83.9-1.00) |  | **<0.001** |
| Enteric bacteria | 29/143 | 20.3  (13.3-27.2) |  | 17/58 | 29.3  (16.7-41.9) |  | 19/121 | 15.7  (8.8-22.6) |  | 0.104 |
| Enteric helminths | 32/67 | 47.8  (35.1-60.5) |  | 6/38 | 15.8  (2.9-28.7) |  | 18/24 | 75.0  (55.6-94.4) |  | **<0.001** |
| Enteric protozoa | 58/67 | 85.6  (77.7-95.5) |  | 14/38 | 36.8  (20.2-53.5) |  | 18/24 | 75.0  (55.6-94.4) |  | **<0.001** |
| Trichomonads | 18/28 | 64.3  (44.8-83.9) |  | NT | NT |  | 2/8 | 25.0  (0.0-61.3) |  | 0.102 |

MTBC: *Mycobacterium tuberculosis* complex; SFV: Simian Foamyvirus; NT: Not tested.

* Prev. (95% C.I.): Prevalence and 95% Wald confidence intervals for a binomial proportion with continuity correction

** Pearson's Chi-squared test comparing the homogeneity of proportions between contexts with simulated p-value based on 2,000 replicates
