# Supplementary material for: Diversity and prevalence of zoonotic infections at the animal-human interface of primate trafficking in Peru
Source: PLoS One. 2024 Feb 7;19(2):e0287893. doi: 10.1371/journal.pone.0287893 (PMC10849265; doi:10.1371/journal.pone.0287893)
Supplement: S2 Table — This table shows the Sorensen dissimilarity indexes obtained through pairwise comparisons of the parasite communities composition found at different context and different monkey genera. (DOCX) [file pone.0287893.s007.docx]

Table S7: Pairwise dissimilarities of parasite communities between contexts for animal-human interaction and host genera of trafficked monkeys

|  | | | | | |  |  |
| --- | --- | --- | --- | --- | --- | --- | --- |
| **Factor** | **Category 1** | **Category 2** | **SOR** | **SIM** | **% turnover** | **SNE** | **% nestedness** |
| **Context** | Captivity | Pet | **0.313** | 0.267 | 85.3% | 0.046 | 14.7% |
|  | Pet | Trade | **0.167** | 0.118 | 70.7% | 0.049 | 29.3% |
|  | Trade | Captivity | **0.235** | 0.133 | 56.6% | 0.102 | 43.4% |
|  |  |  |  |  |  |  |  |
| **Host genera** | Saguinus/leontocebus | Alouatta | **0.818** | 0.750 | 91.7% | 0.068 | 8.3% |
|  | Saimiri | Saguinus/leontocebus | **0.727** | 0.250 | 34.4% | 0.477 | 65.6% |
|  | Aotus | Alouatta | **0.692** | 0.667 | 96.3% | 0.026 | 3.7% |
|  | Lagothrix | Saguinus/leontocebus | **0.652** | 0.000 | 0.0% | 0.652 | 100.0% |
|  | Ateles | Saguinus/leontocebus | **0.625** | 0.250 | 40.0% | 0.375 | 60.0% |
|  | Aotus | Saguinus/leontocebus | **0.600** | 0.500 | 83.3% | 0.100 | 16.7% |
|  | Saguinus/leontocebus | Sapajus | **0.571** | 0.250 | 43.7% | 0.321 | 56.2% |
|  | Aotus | Ateles | **0.556** | 0.333 | 60.0% | 0.222 | 40.0% |
|  | Cebus | Saguinus/leontocebus | **0.538** | 0.250 | 46.4% | 0.288 | 53.6% |
|  | Aotus | Lagothrix | **0.520** | 0.000 | 0.0% | 0.520 | 100.0% |
|  | Aotus | Saimiri | **0.500** | 0.000 | 0.0% | 0.500 | 100.0% |
|  | Aotus | Sapajus | **0.500** | 0.333 | 66.7% | 0.167 | 33.3% |
|  | Ateles | Alouatta | **0.474** | 0.286 | 60.3% | 0.188 | 39.7% |
|  | Aotus | Cebus | **0.467** | 0.333 | 71.4% | 0.133 | 28.6% |
|  | Lagothrix | Alouatta | **0.462** | 0.000 | 0.0% | 0.462 | 100.0% |
|  | Saimiri | Alouatta | **0.440** | 0.000 | 0.0% | 0.440 | 100.0% |
|  | Lagothrix | Sapajus | **0.379** | 0.100 | 26.4% | 0.279 | 73.6% |
|  | Cebus | Alouatta | **0.375** | 0.286 | 76.2% | 0.089 | 23.8% |
|  | Cebus | Lagothrix | **0.357** | 0.000 | 0.0% | 0.357 | 100.0% |
|  | Saimiri | Sapajus | **0.357** | 0.100 | 28.0% | 0.257 | 72.0% |
|  | Ateles | Cebus | **0.333** | 0.222 | 66.7% | 0.111 | 33.3% |
|  | Ateles | Saimiri | **0.333** | 0.167 | 50.0% | 0.167 | 50.0% |
|  | Cebus | Saimiri | **0.333** | 0.000 | 0.0% | 0.333 | 100.0% |
|  | Alouatta | Sapajus | **0.294** | 0.143 | 48.6% | 0.151 | 51.4% |
|  | Ateles | Sapajus | **0.273** | 0.200 | 73.3% | 0.073 | 26.7% |
|  | Cebus | Sapajus | **0.263** | 0.222 | 84.4% | 0.041 | 15.6% |
|  | Ateles | Lagothrix | **0.226** | 0.000 | 0.0% | 0.226 | 100.0% |
|  | Lagothrix | Saimiri | **0.189** | 0.167 | 88.1% | 0.023 | 11.9% |
|  | | | | | |  |  |

SOR: Sorensen dissimilarity index, SIM: Dissimilarity due to turnover, SNE: Dissimilarity due to nestedness.
