# Supplementary material for: Diversity and prevalence of zoonotic infections at the animal-human interface of primate trafficking in Peru
Source: PLoS One. 2024 Feb 7;19(2):e0287893. doi: 10.1371/journal.pone.0287893 (PMC10849265; doi:10.1371/journal.pone.0287893)
Supplement: S3 Table — This table shows the correlation of the different parasite genera with the main two principal components explaining the variation between parasite community composition across contexts for animal-human interaction and host genera of trafficked primates in Peru. (DOCX) [file pone.0287893.s008.docx]

Table S8. Factor loadings of the Principal Components (PC) Analysis.

|  |  | **PC1** | | **PC2** | |
| --- | --- | --- | --- | --- | --- |
|  | Parasite genera* | r | *p-value* | r | *p-value* |
| *Mycobacteria* | *Mycobacteria* | -0.579 | **0.005** | 0.153 | 0.507 |
| *Virus* | *Foamyvirus* | -0.860 | **0.000** | 0.303 | 0.182 |
| *Hemoparasite* | *Dipetalonema* | -0.599 | **0.000** | -0.674 | **0.001** |
|  | *Mansonella* | -0.241 | 0.290 | -0.547 | **0.010** |
|  | *Trypanosoma* | -0.677 | **0.000** | -0.124 | 0.593 |
|  | *Plasmodium* | -0.413 | 0.062 | -0.444 | **0.044** |
| *Enteric bacteria* | *Aeromonas* | -0.156 | 0.497 | 0.268 | 0.240 |
|  | *Campylobacter* | 0.346 | 0.124 | -0.342 | 0.129 |
|  | *Salmonella* | 0.093 | 0.687 | -0.031 | 0.895 |
|  | *Shigella* | -0.112 | 0.629 | 0.547 | **0.010** |
|  | *Plesiomonas* | 0.096 | 0.678 | -0.029 | 0.899 |
| *Enteric helminth* | *Molineus* | -0.606 | **0.003** | -0.460 | **0.036** |
|  | *Prosthenorchis* | -0.598 | **0.004** | -0.674 | **0.001** |
|  | *Strongyloides* | -0.773 | **0.000** | -0.100 | 0.665 |
|  | *Trichuris* | -0.586 | **0.005** | 0.695 | **0.000** |
|  | *Ascaris* | -0.703 | **0.000** | 0.267 | 0.242 |
| *Enteric protozoa* | *Balantidium* | -0.779 | **0.000** | 0.254 | 0.267 |
|  | *Blastocystis* | -0.672 | **0.000** | -0.155 | 0.502 |
|  | *Entamoeba* | -0.628 | **0.002** | -0.111 | 0.632 |
|  | *Cryptosporidium* | -0.656 | **0.001** | -0.086 | 0.712 |
|  | *Giardia* | -0.757 | **0.000** | -0.209 | 0.364 |
| *Trichomonads* | *Dientomoeba* | -0.435 | **0.048** | 0.539 | **0.012** |
|  | *Trichomonas* | -0.528 | **0.014** | 0.652 | **0.001** |

r: Pearson’s correlation coefficient
