# Supplementary material for: Diversity and prevalence of zoonotic infections at the animal-human interface of primate trafficking in Peru
Source: PLoS One. 2024 Feb 7;19(2):e0287893. doi: 10.1371/journal.pone.0287893 (PMC10849265; doi:10.1371/journal.pone.0287893)
Supplement: S4 Table — This table summarizes the generalized linear models (GLM) and generalized linear mixed effects models (GLMM) built to evaluate the contribution of population characteristics to parasite richness. Models ranked by Akaike’s information criterion with small-sample correction (AICc). Statistics include number of parameters (K), log-likelihood (−2LL), difference between AICc of each model and the best model (ΔAICc), and evidence ratio (wi/w1). Models listed under each heading are included in the 95% confidence set. (DOCX) [file pone.0287893.s009.docx]

Table S9. Model selection results for parasite richness among captive primates in Peru. Models ranked by Akaike’s information criterion with small-sample correction (AICc). Statistics include number of parameters (K), log-likelihood (−2LL), difference between AICc of each model and the best model (ΔAICc), and evidence ratio (wi/w1). Models listed under each heading are included in the 95% confidence set.

| **Generalized linear models** | **K** | **-2LL** | **AICc** | Δ**AICc** | **w_i_/w_1_** |
| --- | --- | --- | --- | --- | --- |
| **Including age category (n=372)** |  |  |  |  |  |
| genus + city | 15 | 1047.2 | 1080.7 | 0.0 | 1.00 |
| genus + city + age_category | 17 | 1043.0 | 1080.9 | 0.2 | 0.90 |
| genus + city + context | 17 | 1045.7 | 1083.6 | 2.9 | 0.23 |
| genus + city + age_category + context | 19 | 1041.7 | 1084.1 | 3.4 | 0.18 |
| city | 9 | 1064.0 | 1084.6 | 3.9 | 0.14 |
| city + age_category | 11 | 1059.7 | 1084.6 | 3.9 | 0.14 |
| **Including age category (n=372) and offset** |  |  |  |  |  |
| genus + city + offset (log(mpd)) | 15 | 893.6 | 927.1 | 0.0 | 1.00 |
| genus + city + age category + offset (log(mpd)) | 17 | 891.0 | 928.9 | 1.8 | 0.41 |
| genus + city + context + offset (log(mpd)) | 17 | 893.5 | 931.5 | 4.4 | 0.11 |
| genus + city + age_category + context + offset (log(mpd)) | 19 | 890.8 | 933.2 | 6.1 | 0.05 |
| city + offset (log(mpd)) | 9 | 915.9 | 936.5 | 9.4 | 0.01 |
| city + age_category + offset (log(mpd)) | 11 | 912.8 | 937.7 | 10.6 | 0.00 |
| **Including sex (n=354)** |  |  |  |  |  |
| genus + city | 15 | 1008.1 | 1041.8 | 0.0 | 1.00 |
| genus + city + sex | 16 | 1006.6 | 1042.4 | 0.6 | 0.72 |
| genus + city + age_category + sex | 18 | 1003.3 | 1043.6 | 1.8 | 0.40 |
| genus + city + age_category | 17 | 1005.7 | 1043.7 | 2.0 | 0.38 |
| genus + city + context | 17 | 1006.6 | 1044.7 | 2.9 | 0.23 |
| genus + city + sex + context | 18 | 1004.8 | 1045.0 | 3.3 | 0.19 |
| city | 9 | 1025.5 | 1046.2 | 4.4 | 0.11 |
| genus + city + sex + age category + context | 20 | 1001.5 | 1046.3 | 4.6 | 0.10 |
| **Including sex (n=354) and offset** |  |  |  |  |  |
| genus + city + offset (log(mpd)) | 15 | 856.5 | 890.1 | 0.0 | 1.00 |
| genus + city + sex + offset (log(mpd)) | 16 | 856.3 | 892.1 | 2.0 | 0.37 |
| genus + city + age_category + sex + offset (log(mpd)) | 18 | 854.6 | 894.1 | 4.0 | 0.14 |
| genus + city + age_category + offset (log(mpd)) | 17 | 855.0 | 893.1 | 3.0 | 0.22 |
| genus + city + context + offset (log(mpd)) | 17 | 856.4 | 894.5 | 4.4 | 0.11 |
| genus + city + sex + context + offset (log(mpd)) | 18 | 856.2 | 896.5 | 6.4 | 0.04 |
| city + offset (log(mpd)) | 9 | 880.2 | 900.8 | 10.7 | 0.00 |
| genus + city + sex + age category + context + offset (log(mpd)) | 20 | 854.3 | 899.1 | 9.0 | 0.01 |
|  |  |  |  |  |  |
| **Generalized linear mixed effect models (n=372)** | **K** | **-2LL** | **AICc** | Δ**AICc** | **w_i_/w_1_** |
| genus + city + offset (log(mpd)) + 1/event | 17 | 893.6 | 929.3 | 0.0 | 1.00 |
| genus + city + age category + offset (log(mpd)) + 1/event | 19 | 893.6 | 931.1 | 1.8 | 0.40 |
| genus + city + context + offset (log(mpd)) + 1/event | 19 | 891.0 | 933.7 | 4.4 | 0.11 |
| genus + city + age_category + context + offset (log(mpd)) + 1/event | 21 | 890.8 | 935.5 | 6.2 | 0.05 |
