# Supplementary material for: Diversity and prevalence of zoonotic infections at the animal-human interface of primate trafficking in Peru
Source: PLoS One. 2024 Feb 7;19(2):e0287893. doi: 10.1371/journal.pone.0287893 (PMC10849265; doi:10.1371/journal.pone.0287893)
Supplement: Attachment — Submitted filename: Response to reviewers.docx [file pone.0287893.s010.docx]

June 7th, 2023

Editorial Staff

*PLOS ONE*

Dear Editors,

We resubmit for your consideration the manuscript *“***Diversity and prevalence of zoonotic infections at the animal-human interface of primate trafficking in Peru***”* (PONE-D-23-07657). This resubmission is accompanied with specific responses to the academic editor’s and reviewers’ comments in blue. We thank the reviewers for their thorough reading and helpful critique of our manuscript.

Incorporating the reviewers’ suggestions have added scientific quality as well as clarity and completeness to the manuscript. In its current form, we expect this manuscript to enhance the body of knowledge about zoonotic infections in primates, the risks of primate trafficking to One Health, and to be of particular interest to the readership of your journal.

We thank you very much for your consideration of this manuscript.

Sincerely,

**Ana Patricia Mendoza**

Washington University in St. Louis

Postdoctoral Research Associate

**RESPONSE TO THE EDITOR**

1. Please ensure that your manuscript meets PLOS ONE's style requirements, including those for file naming. The PLOS ONE style templates can be found at [https://journals.plos.org/plosone/s/file?id=wjVg/PLOSOne_formatting_sample_main_body.pdf](https://journals.plos.org/plosone/s/file?id=wjVg/PLOSOne_formatting_sample_main_body.pdf) and 
[https://journals.plos.org/plosone/s/file?id=ba62/PLOSOne_formatting_sample_title_authors_affiliations.pdf](https://journals.plos.org/plosone/s/file?id=ba62/PLOSOne_formatting_sample_title_authors_affiliations.pdf)

Thanks for the reminder. The manuscript style has been revised and updated.

2. We note that you have stated that you will provide repository information for your data at acceptance. Should your manuscript be accepted for publication, we will hold it until you provide the relevant accession numbers or DOIs necessary to access your data. If you wish to make changes to your Data Availability statement, please describe these changes in your cover letter and we will update your Data Availability statement to reflect the information you provide

We will provide DOI for the data repository. We do not need to update Data Availability statement

3. We note that Figure (1) in your submission contain copyrighted images. All PLOS content is published under the Creative Commons Attribution License (CC BY 4.0), which means that the manuscript, images, and Supporting Information files will be freely available online, and any third party is permitted to access, download, copy, distribute, and use these materials in any way, even commercially, with proper attribution. For more information, see our copyright guidelines: [http://journals.plos.org/plosone/s/licenses-and-copyright](http://journals.plos.org/plosone/s/licenses-and-copyright).
 
We require you to either (1) present written permission from the copyright holder to publish these figures specifically under the CC BY 4.0 license, or (2) remove the figures from your submission:
 
1. You may seek permission from the original copyright holder of Figure(s) [#] to publish the content specifically under the CC BY 4.0 license. 
 
We recommend that you contact the original copyright holder with the Content Permission Form ([http://journals.plos.org/plosone/s/file?id=7c09/content-permission-form.pdf](http://journals.plos.org/plosone/s/file?id=7c09/content-permission-form.pdf)) and the following text:
“I request permission for the open-access journal PLOS ONE to publish XXX under the Creative Commons Attribution License (CCAL) CC BY 4.0 ([http://creativecommons.org/licenses/by/4.0/](http://creativecommons.org/licenses/by/4.0/)). Please be aware that this license allows unrestricted use and distribution, even commercially, by third parties. Please reply and provide explicit written permission to publish XXX under a CC BY license and complete the attached form.”
 
Please upload the completed Content Permission Form or other proof of granted permissions as an ""Other"" file with your submission. 
 
In the figure caption of the copyrighted figure, please include the following text: “Reprinted from [ref] under a CC BY license, with permission from [name of publisher], original copyright [original copyright year].”
 
2. If you are unable to obtain permission from the original copyright holder to publish these figures under the CC BY 4.0 license or if the copyright holder’s requirements are incompatible with the CC BY 4.0 license, please either i) remove the figure or ii) supply a replacement figure that complies with the CC BY 4.0 license. Please check copyright information on all replacement figures and update the figure caption with source information. If applicable, please specify in the figure caption text when a figure is similar but not identical to the original image and is therefore for illustrative purposes only.

Figure 1 has been exclusively created for this manuscript and is not under copyright anywhere else. The color layer used as background in the map included as Figure 1 was obtained from the following source:

**Elevation Digital Elevation Model** – Raster layer of global elevations derived from the shuttle Radar Topography mission (SRTM).	https://www.usgs.gov/centers/eros/science/usgs-eros-archive-digital-elevation-shuttle-radar-topography-mission-srtm-1-arc?qt-science_center_objects=0#qt-science_center_objects

Please let us know if there are further problems with this figure.

**Reviewer #1:**

It is an original contribution, the document should define and clarify the wording according to the type of parasite and it is evident that in the wording there is a mixture of parasite types and taxonomic categories.

We thank the reviewer for the careful reading of our manuscript. We have clarified the wording and standardized the terms along the manuscript.

The use of the term “parasite” to represent different parasite types is defined at the beginning of the **Statistical analysis** section of the **Methodology** **(lines 167-171)** and reads as follows:

“All the infectious agents we tested for are referred to as ‘parasites’ because the presence of clinical disease was not investigated (63). Parasite prevalence was estimated as the number of infected individuals over the total number of individuals evaluated for each parasite taxa and parasite type (i.e., mycobacteria, viruses, hemoparasites, enteric bacteria, enteric helminths, enteric protozoa, and trichomonads).”

As in the second sentence of the above paragraph, when pertaining, the use of “parasite” has been replaced by “parasite taxa”

In Table 2, in the values of total samples per genus, primate species, the samples analysed per species should be presented, avoiding presenting the results as total samples per genus.

The number of samples analyzed per species has been added into Table 2.

The discussion is encouraged to be based on the analysis of prevalences and diversities by species and not only by primate genus.

We appreciate this suggestion. However, the discussion of prevalence and diversity at species level falls outside the goals of the manuscript. Please note that the taxonomy of South American primates is changing, and some former species now correspond to multiple taxa. For example, species previously identified as *Cebus albifrons* in Peru now correspond to whether *Cebus yuracus, Cebus cuscinus,* or *Cebus unicolor*. We analyze the results by primate genus as it provides the highest level of taxonomic certainty in our study population.

**Reviewer #2:**

This is a remarkable study of the zoonotic parasites in trafficked primates in Peru. It is an outstanding sampling, diagnosis and analytical effort. The manuscript is clear, well written, organized, an the results are highly relevant for primate parasitology, primate conservation and public health; thus, I suggest publication. There are just a few minor details that I enlist below:

We thank the reviewer for the careful reading of our manuscript, for considering it relevant, and for their time in providing detailed suggestions. We respond to each of their suggestions below:

Line 71: maybe “infectious diseases”

The term has been changed. The text now reads:

“Non-human primates are also susceptible to infectious disease caused by human-associated parasites.”

Line 74: since authors are talking about parasite richness I recommend “number of parasite taxa” to avoid confusion with abundance estimates.

Parasite species richness (PSR) has been replaced by “parasite richness (PR)” along the manuscript.

Lines 179 -180: “parasite taxa” referring to my previous comment.

The term has been included. The text now reads:

“The number of parasite taxa detected by each test was used to estimate the ‘maximum possible detection’ (MPD). MPD = the number of parasite taxa that could have been detected by all the tests carried out on samples from the same monkey.”

Line 199: maybe mentioned the R packages employed.

The main R packages employed have been cited. The text now reads:

“All statistical analyses were performed using R 4.0.3 (R Development Core Team, 2021), RStudio (RStudio Team, 2023) the *vegan* (66), the *DescTools* (67), the *betapart* (65), the *factoextra* (68), the *glmulti* (69), and the *lmer4* (70) packages.”

Lines 218 and 222: “31 parasite taxa”, and “32 parasite taxa”. To avoid confusion with abundance

The term has been replaced. The text now reads:

“Up to seven tests were carried out on samples from the same monkey (median number of tests=3.5, IQR=2.0-5.0) reaching a maximum possible detection of 31 parasite taxa (median MPD=17, IQR=13.0-24.0). […] A total of 1,313 tests were performed, resulting in the detection of 32 parasite taxa.”

Lines 294-313: It is important to mention somewhere in this paragraph, that even though you found in monkeys parasites that can also infect humans, the direction of transmission either monkey-human or human-monkey remains to be determine. Because, as you mentioned later in the discussion, what prevents a monkey to get infected by a human parasite from their captor or owner or keeper?

We thank the reviewer for this important observation. Bidirectional transmission of zoonotic parasites is now mentioned in the paragraph and reads as follows:

“At markets, live monkeys are often observed in the proximity of poultry and raw meat, while pet monkeys are often in close direct contact with children and vulnerable adults within the family environment (8,9). Although infected monkeys may have acquired zoonotic parasites from humans or other infected animals while in captivity, their presence in markets and households represents an additional source of contamination and exposure to zoonotic pathogens at these scenarios.”

Finally, I strongly suggest the authors to define either in the introduction or in methods the way their employing the term “zoonotic”. It is clear to me that they refer to transmission in both directions; but nowadays this term is used in different contexts, for example, the WHO define it as “an infectious disease that has jumped from a non-human animal to humans”, while the CDC states “Zoonotic diseases (also known as zoonoses) are caused by germs that spread between animals and people”. Thus a clear definition would guide the reader to the approach that authors are using in the study

We appreciate this important suggestion. The definition of zoonoses has been included in the abstract and first paragraph of the introduction, and reads as follows:

“Of highest concern among infections that can spread through wildlife trade and traffic are the zoonoses – those infections caused by parasites that can be transmitted between humans and other animals.”

**Reviewer #3:**

This manuscript is aimed to compilate the information on pathogens infecting non-human primates from three different contexts (captivity, households, and trade) in nine locations in Peru, through a cross-sectional study carried out between 2010 and 2012. The subject of the manuscript is relevant on the primate parasitology topic and in the One-Health perspective. This manuscript fully corresponds to the scope of the journal. I consider that the manuscript needs some modifications, clarifications, and corrections before being considered for publication, as well as a general English revision. Some commentaries and suggestions are described below:

We thank the reviewer for its careful reading of the manuscript and their suggestions to improve it. We respond to their observations below:

**Title**
The title states “primate trafficking”, however, according to the methods also
scenarios of captivity and pet were screened. Thus, I suggest modifying the title to
make it more accurate.

We thank the reviewer for this important suggestion. As indicated in the methodology, our study included only wild-caught primates, which originate in the traffic.

The origin of pet and captive monkeys in the trade is also described in the third paragraph of the **introduction**, which reads:

“Monkeys offered for sale or obtained illegally as pets are frequently confiscated by local authorities, temporarily held in custody facilities, and ultimately euthanized or transferred to government-regulated zoos and rescue centers”

Traffic origin is also mentioned in the first paragraph of the **Methods,** under **Study design and sampling strategy**, which reads:

“We sampled wild-caught monkeys at zoos, rescue centers, households, wildlife markets, and temporary custody facilities and classified them into three distinct ‘contexts’ where animal-human interaction occurs: 1) captivity (zoos and rescue centers); 2) pet (households); and 3) trade (wildlife markets or seized during transport to commercial establishments). Monkeys confiscated by local authorities or voluntarily surrendered were assigned to the context they came from (trade or pet) and sampled within the first week of their arrival at the custody facility. To ensure that only animals originated in the primate trafficking were represented in the study, monkeys born in captivity were excluded.”

**Abstract**
- Line 35: “32 parasite taxa” instead of “32 parasites”.

The term has been replaced, now the text reads:

“We detected 32 parasite taxa including…”

- Line 38: as not all parasites were identified at the species level, I would suggest
changing “parasite species richness” to “parasite richness”/ “parasite taxa richness”
along the manuscript.

Parasite species richness (PSR) has been replaced by “parasite richness (PR)” along the manuscript.

- Line 43: “humans” instead of “human”.

The correction has been made. The text now reads:

“Our findings illustrate that the threats of wildlife trafficking to One Health encompass exposure to multiple zoonotic parasites well-known to cause disease in humans, monkeys, and other species.”

-Please include some details about the methods used for parasite identification
(e.g. light microscopy, bacteriological culture, and PCR), and type of samples
collected.

Laboratory techniques are listed in **Table 1**. Detailed procedures for each technique can be found in the publications referenced also in Table 1.

**Key words**
- According to the international primatological community, the term “Neotropical
primates” should be avoided because of the colonial overtones. Please modify it.
Introduction

Although we disagree with the reviewer, the term Neotropical has been removed from most of the manuscript. Please note we used Neotropical as reference to the geographic region, the Neotropics. We avoided the use of “American primates” as it may be misleading for some readers and have used the term “platyrrhine monkeys” although we recognize readers without a primatological background may be unfamiliar with primate parvorders.

- Line 78: delete “all of them platyrrhine monkeys”.

The phrase has been deleted.

- Line 88: 320 parasite species? Parasite taxa? Please specify.

The term “parasites” has been replaced by “parasite taxa”

- Include abbreviation for “non-human primates” along the document.

The abbreviation has been included in **Line 69**. Now it reads:

“As a result of their interaction with us, captive non-human primates (NHP) acquire human-associated parasites…”

- Please include some specific details about previous reports of parasites infecting
non-human primates in Peru. If available, also include the parasite’s prevalence, as
it is a topic dealt with in this manuscript.

We have included information about parasites previously reported in Peru and some prevalence data in the introduction. The fourth paragraph of the **introduction** now reads:

“More than 320 parasite taxa have been reported in monkeys across the Americas, encompassing various types such as enteric helminths, enteric protozoa, hemoparasites, viruses, bacteria, and ectoparasites (40–44). Among these, at least 74 taxa are known to infect humans (42,43). Focusing on zoonotic or potentially zoonotic parasites, studies in Peruvian wild and captive monkeys have detected infections with hemoparasites belonging to the genera *Trypanosoma, Plasmodium, Dipetalonema,* and *Mansonella* (45–51), enteric helminths such as *Ancyclostoma* sp., *Ascaris* sp., *Strongyloides spp., Trypanoxiuris sp., Trichuris trichiura,* and *Schistosoma mansoni.* (52–55), and enteric protozoa of the genera *Blastocytis, Balantidium, Cryptosporidium, Coccidia, Eimeria* and *Entamoeba* (54–57). In the northern Peruvian Amazon, the zoonotic bacteria *Campylobacter spp.* were reported in 21% wild monkeys and 32% pet monkeys (58), while primate rescue centers in the same region report primates infected with antimicrobial-resistant enterobacteria such as *Escherichia coli, Salmonella arizonae, Shigella sp., and Serratia spp.* (41). Within the country, human-associated infections such as mycobacteria (59) and human herpesvirus type 1 (60) have been documented exclusively in captive primates, whereas a high seroprevalence of arbovirus antibodies has been observed solely in wild monkeys (61,62).”

**Methods**
- Lines 118-122: this information is more suitable for the introduction section.

We appreciate the suggestion. The following text has been moved from methods to the third paragraph of the introduction:

“Monkeys offered for sale or obtained illegally as pets are frequently confiscated by local authorities, temporarily held in custody facilities, and ultimately euthanized or transferred to government-regulated zoos and rescue centers (34,35)”

- Table 1: “Dientamoeba” instead of “Dientomoeba”

The term has been corrected.

- Line 145: “thin” instead of “think”

The term has been corrected.

- Line 159: it is stated only “captive monkeys”, please specify if you are referring
also to the other animal-human contexts.

The text has been modified and now reads:

“Table 1. Sampling, storage, and laboratory technique used for the detection of zoonotic parasites in captive, pet, and traded monkeys in Peru”

- Line 169: “for each parasite taxa” instead of “for each parasite”

The term has been replaced, now the text reads:

“Parasite prevalence was estimated as the number of infected individuals over the total number of individuals evaluated for each parasite taxa and parasite type”

- Line 174: “number of parasite taxa” instead of “number of parasites”.

The term has been replaced, now the text reads:

“Parasite richness (PR) was estimated as the number of parasite taxa detected in each monkey by context…”

- Line 184: “context” instead of “contexts”

The term has been corrected.

**Results**
- Line 208: “including” instead of “covering”

The term has been replaced. Now it reads:

“Samples were obtained from 388 monkeys including 18 species and 12 genera…”

- Table 2: “cassiquiarensis” instead of “cassiquairensis”, “parasite richness”/
“parasite taxa richness” instead of “parasite species richness (PSR)”.

The terms have been corrected.

- Line 222: “parasitesº”? or parasite taxa?. Please confirm.

The term has been replaced. The text now reads:

“Up to seven tests were carried out on samples from the same monkey (median number of tests=3.5, IQR=2.0-5.0) reaching a maximum possible detection of 31 parasite taxa (median MPD=17, IQR=13.0-24.0). […] A total of 1,313 tests were performed, resulting in the detection of 32 parasite taxa.”

- Table 3: Please modify the terms and abbreviations for “OWM: Old World
Monkeys” and “NP: Neotropical primates”

“OWM: Old World Monkeys” has been replaced by “catarrhines” and “NP: Neotropical primates” has been replaced by “platyrrhines” along the document.

- Line 260: “nine parasite taxa” instead of “nine parasites”.

The term has been replaced. The text now reads:

“A maximum of nine parasite taxa were detected in the same individual...”

**Discussion**
- Line 288: please modify this sentence “widespread zoonotic infections are a daily threat” as the sampling was conducted more than ten years ago, the actualsituation could be different.

The text now reads:

“Our results illustrate that well-known, widespread zoonotic infections can be a daily threat at the animal-human...”

- Lines 294-297: this information is more suitable for the introduction section.

The text has been removed from the discussion.

- Line 403: “parasite taxa” instead of “parasite species”.

The term has been replaced. The text now reads:

“at least 21 parasite taxa were found in squirrel monkeys…”

- Lines 393-403: please add some information on previously reported parasites
infecting free-ranging monkeys in Peru.

We thank the reviewer for such an important suggestion. References of previous studies in Peru have been added. The text now reads:

“The zoonotic parasites identified in this study show an apparent higher host affinity for squirrel monkeys and wooly monkeys. While further research with larger sample sizes is necessary to confirm this effect, previous studies conducted in Peru on captive and free-ranging monkeys have reported similar trends. Higher prevalence and intensity of filarial infections were observed in squirrel monkeys, wooly monkeys, and gracile capuchins when compared to other free-ranging monkeys hunted for subsistence in the northern Peruvian Amazon (45). Squirrel monkeys, wooly monkeys, and gracile capuchins showed a higher richness of enteric helminths and protozoa among monkey species evaluated at a Peruvian zoo (124). Similarly, infections with enteric helminths and protozoa were more frequent in free-ranging wooly monkeys than in owl monkeys sharing the same forest patch (57). Apart from taxonomic genus, other host characteristics (i.e., sex and age) did not have an effect in our PR model.”

- Line 448: “identified” instead of “identify”.

The term has been replaced. The text now reads:

“We identified several pathogens of public health relevance in trafficked monkeys…”
