# Supplementary material for: Diversity and prevalence of zoonotic infections at the animal-human interface of primate trafficking in Peru
Source: PLoS One. 2024 Feb 7;19(2):e0287893. doi: 10.1371/journal.pone.0287893 (PMC10849265; doi:10.1371/journal.pone.0287893)
Supplement: Attachment — Submitted filename: Response to reviewers.docx [file pone.0287893.s011.docx]

**Reviewer #1:**

A particular aspect that warrants consideration is the scarcity of information regarding the prevalence of Plasmodium malariae/Plasmodium brasilianum infections on a global scale and in the Amazon basin, despite the anticipation of a low prevalence and minimal health impact. Therefore, it is essential to thoroughly explore this subject (see line 336), taking into account the zoonotic nature of these species but not other human malaria species such as Plasmodium falciparum and Plasmodium vixax.

Please, also correct Trypanosoma minasense in line 374.

We thank the reviewer for the careful reading of our manuscript. We have included the information required and corrected the typo mentioned by the reviewer.

Regarding the reviewer’s concern about further investigation in *Plasmodium malariae/Plasmodium brasilianum* – This is a topic that certainly requires further investigation. We are preparing a manuscript discussing the circulation of this parasite in captive facilities in Peru. The potential of this parasite for zoonotic transmission is discussed in more detail in that additional manuscript. The current manuscript was intended to go over the broader range of pathogens infecting captive primates, and so it has not expanded on malaria.
